# Multidisciplinary Management of Carotid Body Tumors in a Tertiary Urban Institution

**DOI:** 10.1155/2015/969372

**Published:** 2015-12-09

**Authors:** George Galyfos, Ioannis Stamatatos, Stavros Kerasidis, Ioannis Stefanidis, Sotirios Giannakakis, Georgios Kastrisios, Georgios Geropapas, Gerasimos Papacharalampous, Chrisostomos Maltezos

**Affiliations:** Department of Vascular Surgery, KAT General Hospital, 14561 Athens, Greece

## Abstract

*Objective*. Aim of this study is to present the experience of our institution in carotid body tumors (CBTs) treatment.* Methods*. All cases treated in a Vascular Surgery Department within 2.5 years (03/2013–09/2015) were retrospectively evaluated. Demographics, diagnostic, and treatment strategy were recorded. All patients with known CBT underwent ultrasound and magnetic resonance imaging preoperatively. All cases were classified according to the Shamblin type and evaluated by a radiologist, otolaryngologist, and anesthesiologist before and after surgery. Major outcomes included mortality, stroke, cranial nerve injury, and recurrence.* Results*. Overall, nine patients (mean age: 59.5 ± 16.3 years) with a total of ten CBTs were treated. There was no gender prevalence and most of the cases (55%) were asymptomatic. There were no functional or familial cases. There was only one bilateral case treated in a staged manner. No preoperative embolization of CBTs was performed. Mortality and stroke rates were null. No severe complication was observed in the early and late setting. No malignancy was recorded. Mean follow-up was 15.6 ± 7.8 months.* Conclusions*. Multidisciplinary management of patients with CBTs is imperative for optimal results, especially in type III tumors, bilateral or functional cases. After careful treatment planning and intraoperative manipulations, complications could be avoided even without preoperative embolization.

## 1. Introduction

Carotid body tumors (CBTs) are the most common tumors of extra-adrenal chromaffin tissue and represent more than 50% of head and neck paragangliomas [1]. However, their incidence is low, reaching only 1 : 30000 [2]. Although these tumors are mostly benign, 5–30% of cases present with a clinical malignant behaviour or increased functionality [3, 4]. Furthermore, due to a potentially infiltrating and disseminating growth, CBTs should be regarded as semimalignant. Therefore, early detection, proper investigation, and effective treatment are imperative.

Aim of this retrospective study is to present a small but significant cohort of CBTs that were managed and treated in our institution. Epidemiologic data, diagnostic investigation, and therapeutic management was recorded and presented. Finally, data are compared to international literature in order to produce useful conclusions.

## 2. Materials and Methods

We retrospectively evaluated all patients treated for a CBT during a period of almost 2.5 years (03/2013–09/2015) in our institution. All patients were treated in the Vascular Surgery Department of a tertiary urban general hospital. All epidemiologic data of patients, clinical presentation, and imaging diagnostics were recorded.

All patients with a known CBT before surgery underwent ultrasound and magnetic resonance (MR) imaging in order to classify the type of CBT and plan the proper treatment. All CBTs were classified according to MR criteria by an experienced radiologist [5]. The classification was further verified during the postoperative histopathological examination, based on the Shamblin classification [6]. This system classifies the tumors into three groups: Group I (tumors are too small and do not involve the surrounding vessels), Group II (tumors are adherent or partially surround and compress the carotid vessels without being problematic for resection), and Group III (tumors show an intimate adherent relationship to the entire circumference of the carotid bifurcation, requiring partial or complete vessel resection and reconstruction). Before surgery, all patients were examined by an otolaryngologist and anesthesiologist as part of a multidisciplinary preoperative management. Finally, in cases suspicious for functional tumours (typical symptoms such as flushing, palpitations, or headaches), the patients underwent a full blood testing for levels of catecholamines, thyroid hormone, and other types of hormones.

All cases underwent open repair under general anesthesia. Primary goal of the surgery was to extract the CBT without further manipulating the carotid vessels, unless it was warranted. Resection was performed through a longitudinal cervical incision made along the anterior border of the sternocleidomastoid muscle. Intraoperatively, the internal jugular vein was identified and the common facial vein ligated. The common carotid artery was dissected to obtain proximal control. The technique used involved subadventitial or sometimes periadventitial resection. Cutting into the tumor was sometimes reserved for the advanced Shamblin classes to free the carotid vessels of the encircling tumor and finally secure the feeders. The hypoglossal nerve, the vagus nerve, and sometimes the glossopharyngeal nerve were identified away from the tumor and, if infiltrated, gradually and meticulously dissected and freed if possible.

Early as well as later complications of the procedure were evaluated and recorded. Major outcomes included mortality, stroke, and cranial nerve injury as well as late recurrences. Postoperative laryngoscopy plus phoniatrics evaluations were ordered in all cases. All patients were treated by the same head surgeon. Clinical evaluation and ultrasound examination followed at 6 months as well as one year after each procedure.

## 3. Results

**Figure 1:**
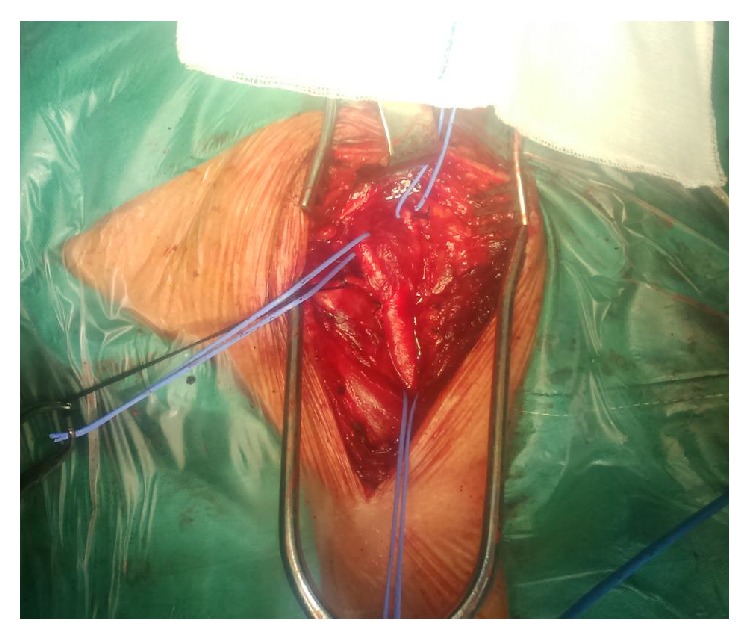
Intraoperative image showing a carotid artery bifurcation and a type II carotid body tumor.

During the overall period of 2.5 years, nine patients with a total of 10 CBTs were treated by surgical excision in our institution. There were 5 women (55%) and 4 men (45%) treated, with an overall age ranging from 19 to 78 years (mean age: 59.5 ± 16.3 years). Regarding clinical presentation, only one (11%) patient had presented with bilaterally located CBTs. Out of 9 patients, three (33%) patients had complaints of a painless mass in the lateral cervical region, five (55%) patients had an incidental diagnosis by an ultrasound evaluation during investigation for other reasons, and finally, in one patient, the CBT was accidentally discovered during an endarterectomy for carotid artery stenosis. The latter patient had undergone only ultrasound and digital angiography before CEA that had not identified the presence of a small CBT. All other cases with a known CBT before surgery underwent a MR examination to classify the tumor (Figure 1). No other complications such as nerve palsy, dysphagia/dysphonia, flushing, or palpitations were reported before excision. Finally there were no familial cases reported when medical history of patients was obtained (Table 1).

**Figure 2:**
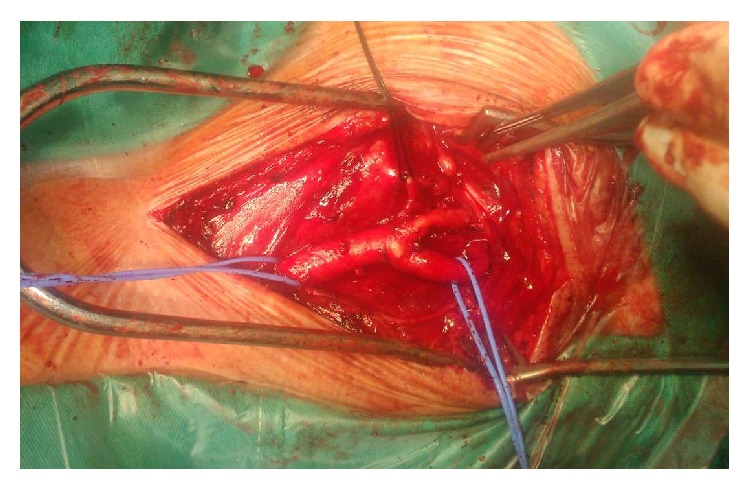
Intraoperative image showing the intact carotid bifurcation after the excision of a carotid body tumor.

**Figure 3:**
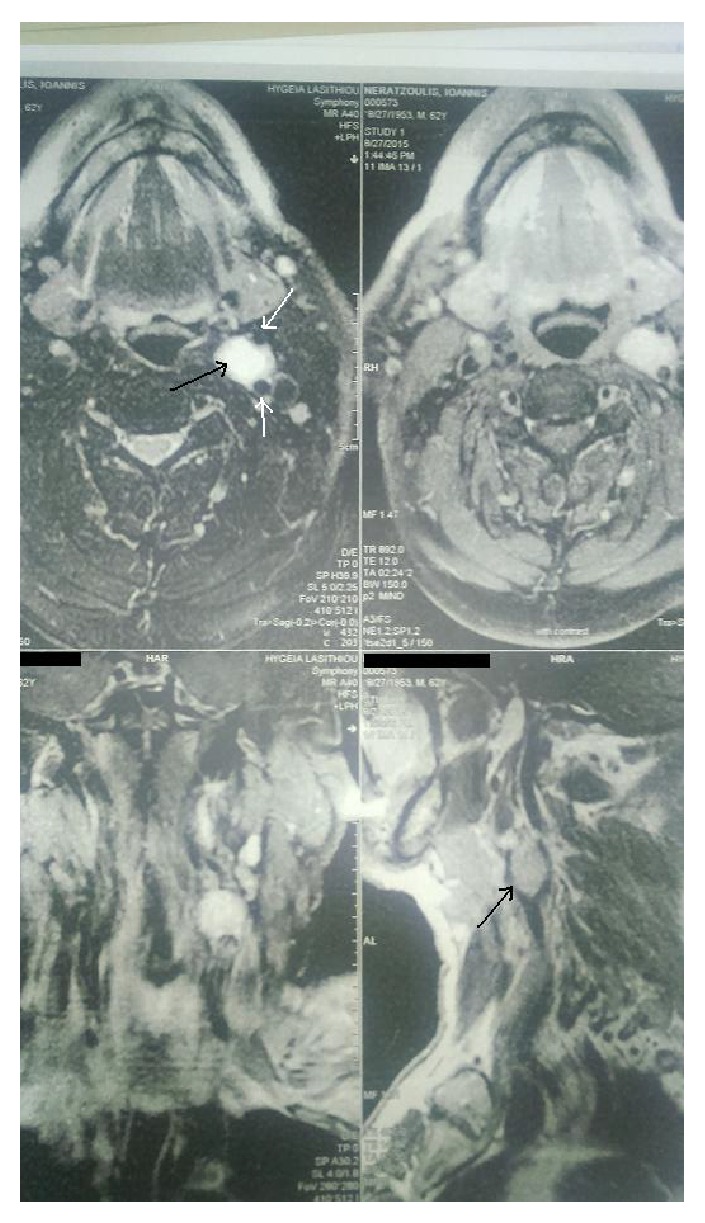
Magnetic resonance image showing a type II carotid body tumor (black arrows) that partially surrounds and compresses the carotid vessels (white arrows).

According to the Shamblin classification, there were 4 (40%) CBTs of type I, 4 (40%) CBTs of type II, and 2 (20%) CBTs of type III identified. Half of the CBTs were located on the left side and half of them on the right side. There was no preoperative embolization of CBTs performed, even in the cases of larger size. In one case of bilateral CBTs, the tumors were removed in staged procedures, with the repair of the smaller CBT scheduled three months after the excision of the larger CBT. Only in the two cases with type III CBT, a partial resection of internal carotid artery was needed to be performed in order to remove the firmly adhered CBT, and an interposition of a great saphenous vein graft (end-to-end anastomoses) followed (Table 2). Intraoperatively, two patients presented bradycardia that needed to be reversed using atropine. No severe hypotension was recorded (Table 3) (Figures 2 and 3).

Regarding major outcomes, there was no operative mortality, the perioperative stroke rate was null, and no revision was needed for bleeding. There were no peripheral neurologic complications as well. No patient showed any dysphonia, dysphagia, or dysgeusia postoperatively. All resected lymph nodes were negative for malignancy. All pathologic specimens were diagnosed as paragangliomas of the carotid artery. Mean follow-up lasted for 15.6 ± 7.8 months. No midterm complications or tumor recurrence was observed during follow-up either (Table 3).

## 4. Discussion

In this retrospective study, we have presented a small but significant series of CBTs treated in a multidisciplinary manner with satisfying results. All patients were treated with primary surgery without preoperative embolization. Perioperative mortality was null, with no postoperative complications observed, both in the early and in the late setting. No recurrence was recorded either.

Regarding the epidemiology, the usual age onset is 20 to 60 years, except when there is a genetic predisposition [7]. In such cases, the incidence of familial CBTs reaches almost 30% [2]. Indeed, paraganglioma patients have been associated lately with succinate dehydrogenase- (SDH-) gene mutations and, thus, all of them should undergo screening testing in order to design further treatment [8]. However, in our series, there was only one 19-year-old patient, with no familial history of CBTs being reported. Moreover, the prevalence of CBTs showed no gender difference in our cohort, concurring with the literature [3]. In general, the most frequent clinical presentation reported is a palpable painless cervical mass although only one-third of our cases reported such a symptom and the diagnosis was incidental in more than a half of our patients. Other less frequent symptoms include flushing, palpitations, or headaches suggestive of a functionally active tumor. The patient may present with symptoms of dysphagia, choking, or hoarseness depending upon the cranial nerve involvement [9].

Concerning preoperative diagnostic evaluation, Duplex ultrasound scan is able to provide information such as tumor size, its position and relationship with adjacent structures, and intralesional blood flow signals [10, 11]. Given that this is a noninvasive and readily available examination, it remains the first-line imaging modality for the assessment of CBTs. For further investigation, computed tomography or MR studies provide details concerning the tumor characterization and the regional extension of CBTs and allow for tumor volume assessment [12, 13]. The classic “salt and pepper” imaging appearance of these lesions is observed on T2 weighted images, where the “pepper” refers to the low signal flow voids and the “salt” refers to high signal foci of hemorrhage and/or slow flow [14]. Moreover, both modalities can predict the Shamblin classification, according to several authors [5, 13]. Therefore, both ultrasound and MR imaging were ordered for all our patients. Finally, selective digital angiography can identify the feeding vessels of the tumor, usually originating from the external carotid artery. Although it is considered to be the golden standard for diagnosis by some authors [8, 15], no CBT was detected on the digital angiography performed in our patient who underwent CEA. Additionally, an invasive examination such as digital angiography should be recommended only when embolization is planned to be performed [10, 12]. Finally, several authors have recently highlighted the promising results of 68-gallium-1,4,7,10-tetraazacyclododecane-1,4,7,10-tetraacetic acid-1-Nal3-octreotide ((68)Ga-DOTA-NOC) positron emission CT tomography (PET-CT) in detection and characterization of paragangliomas, although larger studies are needed to verify its performance [16, 17].

The selection of treatment depends on the size, location, and biologic activity of the tumor as well as the overall fitness of the patient. Surgical excision remains the golden standard of therapy due to the risk of local complications related to tumor size and the risk of malignancy [2]. Concerning postoperative outcomes, surgery has been associated with several complications such as stroke, cranial nerve injury, and bleeding. Cranial nerve injury occurs usually in 19% to 49% of patients [18] although none of our cases presented any such symptom. Speech and swallowing difficulties could be produced in the immediate postoperative period, and most of these deficits are compensated with rehabilitation [19]. When a malignancy is suspected, tumor resection should be accompanied by lymphadenectomy and eventual radiotherapy which seems to be effective for isolated metastasis [20], although no malignancy was diagnosed in our series. Finally, when the tumor is considered functional, careful evaluation before surgery, measurement of serum catecholamine, treatment with adrenergic blockers, and gentle manipulations during the excision are essential for optimal results [4].

Several techniques have been proposed to reduce adverse outcomes and to achieve optimal results as well. Visualization and careful dissection of the principal regional nerves (vagus nerve, hypoglossal nerve, and the superior laryngeal nerve) are mandatory [2]. Careful and accurate dissection of the tumor should be performed along the subadventitial plane or “White Line” suggested by Gordon-Taylor [21] in order to separate the tumor through a relatively avascular plane. However, as shown in our study, excision of larger tumors could necessitate carotid segment resection and graft interpositioning. Early external carotid artery division intraoperatively seems to reduce the stroke rate [22] as well as bleeding rate [23], although this was not performed in our patients with satisfying results. Zeng et al. have also recommended standardized shunting during CBT excision in order to exclude the vascular supply of external carotid artery, reduce the size of the tumor, and guide the resection in difficult cases [24]. As recommended by many authors [9, 15, 25] and applied in our institution as well, multidisciplinary approach of diagnosis and treatment including radiologists, vascular surgeons, otolaryngologists, and anesthesiologists is imperative for optimal results.

In bilateral cases, data still remain controversial. In a recent multicenter review of CBT [10] management, 18% of tumors were bilateral although the same rate was lower in our study. The goal of treatment should be the preservation of life quality rather than the cure of the disease itself. Some authors [26, 27] prefer to resect the larger tumor first. The main reason is that if the patient sustains a cranial nerve injury after the first procedure, the contralateral tumor of the other side could be monitored closely without resection or it could be treated by alternative means such as radiotherapy. However, Kruger et al. conclude that the smaller tumor should be resected first, followed by the staged resection of the larger CBT [28]. Finally, baroreflex failure syndrome is a rare but significant complication after bilateral CBT excision including severe fluctuations of blood pressure [29]. In our study, only one case had bilateral CBTs and staged excision was performed without any early or late adverse events.

Proper preoperative classification is imperative for optimal and successful management. The classic Shamblin classification [6] has been proposed over 40 years ago; however, it remains a highly subjective system according to many authors [18]. However, as the definition itself implies, this classification is mainly useful for predicting vascular morbidity rather than major neurological outcomes after surgery [30]. Therefore, type III tumors were associated with arterial resection in our study. Assessment of tumor size, volume, and anatomic relationships to structures, such as the angle of the jaw and the vertebral bodies, may provide a more objective way to predict perioperative morbidity as well [18].

Furthermore, there has been a debate during the last decades regarding the indication of preoperative embolization of CBTs [9]. The aim of such procedure includes preoperative tumor shrinking, reducing vascularization, and intraoperative bleeding risk, especially for CBTs of larger size (Shamblin types II and III) [31]. Such procedure should be undertaken only in those vessels that can be subselectively catheterized and determined not to allow free reflux of contrast medium into the internal carotid artery [32]. In a recent meta-analysis, the authors conclude that surgical resection combined with preoperative embolization decreases blood loss and operative time as well [33]. A large retrospective review of more than 2,000 patients found significantly lower risk for hematoma and transfusion after endovascular treatment, although the cost outweighed open surgery by far [34]. Boscarino et al. recommend performing embolization the day before the surgical procedure in order to avoid any inflammatory reaction around the lesion and to achieve optimal bleeding risk reduction [2]. However, several authors advocate that large CBTs could be equally resected safely both with and without preoperative embolization [18]. Sen et al. have shown that preoperative embolization does not reduce neurological complications, although the rates of stroke and nerve palsy after surgery are acceptable even in large tumors [19]. This concurs with our results where no patient in our cohort underwent any endovascular procedure although the outcome was optimal.

Limitations of our study include the small number of cases presented. However, taking into consideration the number of cases per year, this rate is similar to the ratio of most of the published studies. Additionally, the follow-up period after surgery does not permit long-term conclusions regarding the recurrence of such tumors. Finally, the small number of patients did not allow for subanalysis among the Shamblin groups to be performed.

## 5. Conclusions

Multidisciplinary management of patients with CBTs is necessary to ensure satisfying results and avoid complications, even in demanding cases. After careful treatment planning and intraoperative manipulations, outcomes are optimal even without preoperative embolization.

## Figures and Tables

**Table 1:** Demographic data of patients included in the study.

Variable	Mean values or number
Number of patients	9
Number of tumors resected	10
Age	59.5 ± 16.3 years
Male gender	4 (45%)
Bilateral tumors	1 (11%)
Family history	0
Presentation	
Painless mass	3 (33%)
Flushing/palpitations	0
Incidental discovery	6 (67%)
Syncope/presyncope	0
Painful mass	0
Dysphagia/dysgeusia	0
Dysphonia	0
Amaurosis	0
Imaging	
Ultrasound duplex scan	9 (100%)
Computed tomography	0
Digital angiography	1 (11%)
Magnetic resonance angiography	8 (89%)

**Table 2:** Management of patients included in the study.

Variable	Mean values or number
Preoperative embolization	0
Resection alone	8 (80%)
Prosthetic patch	0
Saphenous graft interposition	2 (20%)
Primary end-to-end anastomosis	0
Shamblin classification	
Type I	4 (40%)
Type II	4 (40%)
Type III	2 (20%)

**Table 3:** Outcomes, complications, and follow-up of patients.

Variable	Mean values or number
Intraoperative	
Death	0
Stroke	0
Cranial nerve resection	0
Severe bradycardia or hypotension	2 (20%)
Major bleeding/vascular injury	0
Operating time (minutes)	113 ± 18
Postoperative	
Respiratory failure	0
Hematoma	0
Reintervention	0
Infection	0
Cranial nerve injury	0
Death	0
Stroke	0
Follow-up (months)	15.6 ± 7.8
Recurrence rate	0
